# Management of acute periprosthetic knee infection: a comparison of arthroscopic and open debridement

**DOI:** 10.1007/s00402-023-04782-5

**Published:** 2023-02-03

**Authors:** A. Bartsch, P. Krenn, B. Lubberts, M. Morgenstern, G. Pagenstert, M. Clauss

**Affiliations:** 1 Department of Orthopaedic and Trauma Surgery, University Hospital Basel, Spitalstr. 21, 4031 Basel, Switzerland; 2 Centre for Musculoskeletal Infections, University Hospital Basel, Spitalstrasse 21, 4031 Basel, Switzerland; 3 Clarahof Praxisgemeinschaft für Orthopädie Basel, Clarahofweg 19a, 4058 Basel, Switzerland

**Keywords:** Periprosthetic joint infection, Knee arthroplasty, Arthroscopic debridement, Open debridement, DAIR

## Abstract

**Introduction:**

In acute periprosthetic knee infections, debridement and implant retention (DAIR) is the preferred treatment prior to one- and two-stage revisions. The aim of this study is to compare the outcomes of arthroscopic and open debridement of infected primary total knee arthroplasties (TKA).

**Material and methods:**

We analyzed clinical, laboratory, and antibiotic treatment data, collected in patients with periprosthetic knee infection treated with DAIR at a Swiss Level 1 orthopedic and trauma center over a 10-year period between January 2005 and May 2015. Inclusion criteria were primary total knee arthroplasty and early postoperative or acute hematogenous periprosthetic joint infection (PJI). The primary endpoint was the need for further revision surgery due to persistent infection. The secondary endpoint was the prosthesis salvage in further infection surgeries.

**Results:**

Forty-two patients with 44 acute or hematogenous periprosthetic knee infections were included. We recorded 20 recurrent infections (45%) in our study population: 10 (77%) out of 13 in the arthroscopic DAIR group and 10 (32%) out of 31 in the open DAIR group. Two-stage revision, meaning complete removal of the TKA, insertion of a spacer and replantation at a second stage, had to be performed in three patients treated initially arthroscopically (23%) and in six patients treated initially with an open surgical procedure (21%).

**Conclusions:**

Open debridement for acute periprosthetic knee infection shows clear benefits in terms of infection eradication and prosthesis salvage compared to arthroscopic DAIR.

## Introduction

The success of surgical treatment of periprosthetic joint infection (PJI) is eminently dependent on a radical debridement of the periprosthetic infected tissue [5]. Debridement, antibiotics, and implant retention (DAIR) is the preferred treatment option in early postoperative (within 4 weeks after arthroplasty) and acute hematogenous periprosthetic infection (duration of symptoms less than 4 weeks) compared to the more invasive one- and two-stage revisions [25].

Open and arthroscopic debridements are both established procedures for removal of the infected tissue. Each procedure has its advantages and disadvantages to be considered for the individual patient and situation. Arthroscopic debridement is a minimal-invasive, simple procedure with higher popularity among non-arthroplasty surgeons, yet it was shown to have unfavorable outcomes in the treatment of acute [14] and chronic infections [32]. With this procedure, limited access to the posterior capsule, the lack of possibility of radical debridement, and no ability to exchange of polyethylene (PE) bearings have been proven to be problematic [3, 4, 14, 32]. In contrast, the open surgical procedure is more invasive and technically more demanding and is therefore favored by experienced arthroplasty surgeons. Aside from a radical debridement, the bacterial biofilm on implant material can be tackled by PE exchange when using this procedure [2, 14, 27].

In case of early postoperative or acute hematogenous infections with an immature biofilm, the question arises whether performing an open procedure is truly necessary or whether an infection can be treated arthroscopically with similar success rate. Kim et al. retrospectively reviewed 28 infected TKAs and compared variables between success and failure groups [17]. They found that retention of the PE liner (as in case of arthroscopic debridement) was a risk factor for failure (*p* = 0.017). Johns et al. compared as the only study directly arthroscopic and open DAIR procedures in acutely infected TKAs and showed a 29% greater success rate for open DAIR (45% open vs 16% arthroscopic; *p* < 0.001), but follow-up was limited to an average of 2.6 years [14]. Despite superior outcome with the open procedure, the successful PJI treatment rate of 45% was still rather low and Johns et al. [14] did not provide further information on their antibiotic treatment.

The aim of this study is to compare the outcomes of patients undergoing arthroscopic or open debridement for early and acute hematogenous periprosthetic knee infection at our institution. We hypothesized that arthroscopic DAIR shows unfavorable results in terms of infection eradication and prosthesis survival compared to open DAIR and expected higher overall success rates compared to the study of Johns [14] due to using a well-established antibiotic treatment protocol.

## Material and methods

We retrospectively analyzed the medical records of all patients who underwent treatment for periprosthetic knee infection at a Swiss Level 1 trauma center over a 10-year period between January 2005 and May 2015. Patients with periprosthetic knee infection were identified through screening of diagnostic coding in our institution’s electronic medical database. Inclusion criteria were either early postoperative (within 4 weeks after arthroplasty) or acute hematogenous (symptoms less than 4-week duration) PJI in patients with primary total knee arthroplasty, who received surgical revision combined with an established antibiotic treatment [36]. Patients were excluded if they had a chronic infection (duration > 4 weeks), if they underwent initial debridement in another hospital or if antibiotic therapy was not completed according to protocol. All surgeries were performed by board certified orthopedic surgeons including all hierarchies. Therapy decision was made by the individual surgeons at their discretion, based on the patient’s condition and the surgeon’s skills. There was no specialized musculoskeletal infection unit for the treatment of PJI established at our institution during the time period of the study.

### Definition of infection

The diagnosis of PJI was established according to the criteria published by Zimmerli et al. [36] and the criteria published in 2021 by the European Bone and Joint Infection Society (EBJIS) [21]. Prior to surgery patients underwent routine serologic testing (C-reactive protein (CRP), white blood cell count (WBC)) and synovial fluid analysis (cell count, cell differential). During revision surgery for PJI, at least three deep tissue samples were collected for both microbiological and histopathological analysis [19]. Patient demographic information (age, sex, tobacco use, diabetes, intravenous drug abuse, and immunomodulating medications, e.g., local steroid injections [1, 24]) was collected from the medical records and documented in an encoded Microsoft Excel spreadsheet (Microsoft Office Professional Plus 2016) (Table 1).

**Table 1:** Confirmatory EBJIS criteria for the diagnosis of PJI used in this study (adapted from McNally et al. [21])

	Infection confirmed
Clinical and blood workup	
Clinical features	Sinus tract with evidence of communication to the joint or visualization of the prosthesis
Synovial fluid	
Leukocyte count (cells/μl)	> 3000
PMN (%)	> 80%
Microbiology	
Intraoperative (fluid and tissue)	≥ 2 positive samples with the same microorganism
Sonication (CFU/ml)	> 50 CFU/ml of any organism
Histology	
Microscopy	Presence of ≥ 5 neutrophils in ≥ 5 HPF
	Presence of visible microorganisms

### Operative therapy

For skin preparation, povidone iodine was applied in all cases. A pneumatic tourniquet was used per surgeons’ preference and varied in both groups.

Arthroscopic debridement was performed through an anteromedial and anterolateral portal using a minimum of 6 L of saline fluid and a motorized shaver for synovectomy.

For open debridement, the preexisting medial parapatellar incision was used for arthrotomy and tissue samples collected prior to radical synovectomy. Afterward, the knee was flushed with bactericidal solutions (Lavasept, Polihexanidum) using a high-pressure pulse lavage. In case of PE exchange, the implant was sent for sonication.

### Antibiotic therapy

All patients received empiric broad-spectrum antibiotic therapy (amoxicillin/clavulanic acid) after harvesting of the samples, which was later substituted by targeted antibiotic therapy according to the antibiogram. The antibiotic therapy was in all cases established in close collaboration with an infectious disease (ID) specialist and was provided for a period of three months following antibiotic treatment initiation in line with the established PJI protocol of Zimmerli et al. [36].

### Outcome parameters

Successful infection treatment was defined as absence of the above mentioned criteria of PJI without any clinical signs of infection at final follow-up. Failure was measured by evaluating the number of recurrent infections and necessary re-interventions from the clinical records. The primary endpoint was the need for revision surgery due to persistent/uncontrolled infection. The secondary endpoint was prosthesis salvage in further infection surgeries.

Chi-squared tests were conducted to examine whether there was a significant difference in surgery success (1) for differences in success between the open and arthroscopic debridement (2) in final TKA salvage between the initial open and arthroscopic debridement (3) for early and hematogenous TKA infections (4) dependent on patients’ risk factors as tobacco use, diabetes, immunosuppression (5) dependent on patients’ gender. In addition, Chi-squared tests were used to find differences between open and arthroscopic DAIR groups, concerning (1) surgery emergency classification (2) performing surgeons (3) attending perioperative intensive care unit.

Logistic regression analysis was conducted to examine whether patients age, time period from TKA implantation to infection occurrence, or parameters of serologic and synovial fluid analysis had influence on surgery success.

Independent *t* tests were performed to find differences between open and arthroscopic DAIR concerning the interval from presentation in the clinic to surgery.

## Results

### Patient population

A total of 48 patients have been treated for suspected acute PJI during the study period. During diagnostic workup, five patients had to be excluded because PJI could not be confirmed [aseptic loosening of the prosthesis (*n* = 2) or other aseptic irritation of the knee (*n* = 3)]. One patient with confirmed PJI refused antibiotic therapy and was excluded as well. Two patients had simultaneous PJI of both knees due to hematogenous infection and received bilateral surgical infection treatment. Thus, 42 patients with 44 infected TKAs were finally eligible for analysis. The patients’ demographic characteristics and risk factors are shown in Table 2. The mean age of patients at the time of PJI diagnosis was 70.7 years (range, 34.4–87.7 years); 52% were male. The follow-up time after revision surgery of each patient was at least 6 years. Mean follow-up time after index surgery was 8.4 years and after last revision surgery 3.8 years (range, 2–9.4 years). No patient had documented active intravenous drug abuse.

**Table 2:** Patient demographic information

Number of patients	Total (*n* = 44)	Arthroscopic DAIR (*n* = 13)	Open DAIR (*n* = 31)	*p* value
Age [mean years] ± SD	70.7 ± 12.2	75.5 ± 13.6	69.2 ± 9.7	0.185
Sex male, *n* (%)	23 (52%)	7 (54%)	16 (52%)	0.929
Tobacco use, *n* (%)	6 (14%)	1 (8%)	5 (16%)	0.45
Diabetes, *n* (%)	18 (41%)	6 (46%)	12 (39%)	0.29
Immunosuppressed, *n* (%)	8 (18%)	2 (15%)	6 (19%)	0.74
Interval from implantation to infection diagnosis* [mean years] ± SD	3.1 ± 4.8	5.4 ± 7.8	2.2 ± 2.3	0.045
PJI type: Early postoperative, *n* (%)	6 (14%)	1 (8%)	5 (16%)	0.21

*DAIR* debridement, antibiotics, and implant retention, *PJI* periprosthetic joint infection, *SD* standard deviation

*Only in the 38 PJIs with acute hematogenous infection

We found a significant difference (*p* = 0.045) for the time interval between TKA implantation and onset of symptoms between open and arthroscopic DAIR. Patients’ age, gender, tobacco use, diabetes and immunosuppression did not correlate between the groups (Table 2).

### Perioperative management

Thirty-nine of the 44 TKAs (89%) were admitted to the hospital through the emergency department. The median time from admission to surgery was 27 h. Arthroscopic DAIR was performed in median 11 h earlier than open DAIR (*p* = 0.532). While 77% of the arthroscopic DAIRs was performed as an emergency surgery (i.e., within < 24 h), open DAIR was more likely to be scheduled in the routine OR program the next day with 39% as emergency classified surgeries (*p* = 0.026). Open DAIR procedures were more likely performed by a senior physician (65% vs 31%, *p* = 0.029). Nineteen of the 44 cases (43%) needed perioperative care on an intensive care unit with no significant differences between groups (*p* = 0.864).

### Laboratory, pathology and microbial culture results

Prior to revision surgery, all patients underwent serologic testing and 24 patients (55%) had prior synovial fluid analysis. Overall, 68% (30/44) TKA had CRP elevated over 100 mg/L. 50% (12/24) had a synovial fluid cell count over 50,000/mm^3^. Detailed preoperative serologic and synovial fluid laboratory findings are shown in Table 3.

**Table 3:** Preoperative laboratory values

	Total	Arthroscopic DAIR	Open DAIR	*p* value
Serum analysis [mean]				
Preoperative CRP level (normal CRP level < 3) [mg/L]	169	229	143	0.121
Preoperative White blood cell count (normal WBC 3.5–10) [× 10^9^/l]	15.1	26.6	10.3	0.570
Synovial fluid analysis [mean]*				
Cell count [/mm^3^]	69,000	96,550	64,970	0.539
Cell count above 3000/mm^3^ [*n* patients]	24	8	16	1.0
Polynuclear cells (%)	90.0	88.8	90.6	0.965

*CRP* C-reactive protein, *DAIR* debridement, antibiotics, and implant retention, *WBC* white blood cell count

*Synovial fluid analysis available in 24 patients

In total 95% (42/44 TKA) had comprehensible intraoperative microbial cultures. In two patients, documentation was missing in the laboratory records or biopsies were not taken intraoperatively. Both patients were treated as infected due to clinical findings. 37/42 patients (88%) had positive intraoperative microbial cultures. In 5/42 (12%) no bacteria could be isolated, but infection was diagnosed due to fistula or purulent drainage. The most common infectious organism was *Staphylococcus* spp (32%) followed by *Streptococcus* spp (25%). Microbial testing results are listed in Table 4. All staphylococcal strains were sensitive to rifampicin.

**Table 4:** Intraoperative culture results

	Total (*n* = 42)	Total arthroscopic DAIR (*n* = 13), (failure %)	Total open DAIR (*n* = 29), (failure %)
*Streptococcus* spp.	11 (25%)	4 (25%)	7 (57%)
*Staph. aureus*	10 (23%)	3 (100%)	7 (71%)
Culture negative	5 (12%)	2 (100%)	3 (0%)
Enterococus spp.	5 (11%)	2 (100%)	3 (0%)
Coagulase-negative *Staphylococc*i	4 (9%)	1 (100%)	3 (0%)
Polymicrobial infection	4 (9%)	1 (100%)	3 (0%)
Gram-negative organisms	3 (7%)	0	3 (33%)

*DAIR* debridement, antibiotics, and implant retention, *spp.* species

### Infection recurrence

**Fig. 1:**
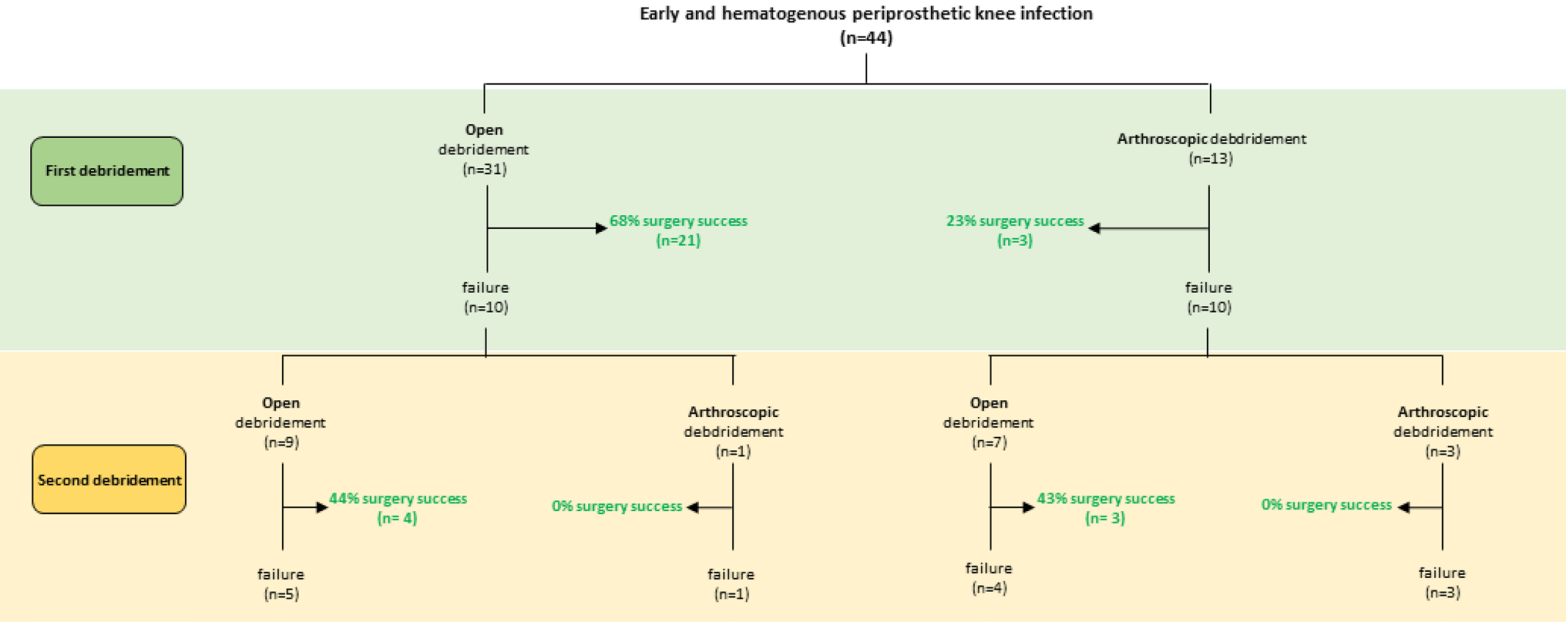
Patient flowchart. Outcome of open and arthroscopic debridement

Thirty-one TKA (70%) initially underwent open DAIR and thirteen TKA (30%) initially underwent arthroscopic DAIR (Fig. 1). In total, 20 out of 44 cases (45%) had persistent infections after initial DAIR. Arthroscopic DAIR showed unfavorable results with ten failures out of thirteen debridements (77%) compared to the open DAIR group with ten failures out of 31 debridements, (32%). This finding was statistically significant (*X*^2^ (*df* = 1, *N* = 44) = 7.37, *p* = 0.007).

During initial open DAIR, 5 of the 31 infected TKAs received a mechanical debridement of the PE but the original cleaned inlay of the infected knee was reimplanted. Of these, three surgeries were successful, and two required a further secondary DAIR. In all other cases the PE was exchanged. The overall failure rate in our open DAIR cohort did not change significantly with or without PE exchange (40% vs 44%, *p* = 0.685).

Of the ten failed initial arthroscopic DAIR cases, three received further arthroscopic debridement, but none could be effectively treated with this procedure and finally received open debridement. Of the ten failed open DAIR cases, one received further arthroscopic debridement, but also could not be successfully treated with this procedure. Success rate for open debridement at the second attempt was approximately 45%, regardless of whether the first debridement was open or arthroscopic (Fig. 1).

Two-stage revisions had to be performed in nine patients in total, of which three patients were treated arthroscopic initially (23%) and six patients were initially treated with an open procedure (21%). There was no significant difference detected in final TKA salvage between initial open and initial arthroscopic debridement group (*X*^2^ (*df* = 1, *N* = 44) = 0.297, *p* = 0.586).

## Discussion

Periprosthetic infections are a delicate matter for both patient and surgeon. The therapy must be well chosen in order to eradicate pathogens sufficiently. Our data suggest that arthroscopic debridement in early postoperative and acute hematogenous periprosthetic infection has significantly lower success rates than open debridement. This confirms previous studies investigating periprosthetic joint infection in the knee [13, 14, 34]. High failure rates for the treatment of periprosthetic knee infections are described in literature following both arthroscopic and open debridement [4, 6, 7, 9, 11, 17, 18, 20, 22, 23, 30–33, 37]. Similarly, our data show high revision rates, with a total of 45% revisions (77% arthroscopic vs. 32% open) after index revision. Even in two-stage revisions, failure rates have been reported approximately 20%, with a wide range from 9 to 50% [8, 10, 12, 24]. One explanation for the variable outcomes in both open and arthroscopic surgery might be the disparate extent and quality of debridement performance among surgeons. In general, arthroscopic debridement does not allow radical debridement and limits access to the posterior soft tissue capsule. Moreover, it does not permit the exchange of polyethylene bearings, which is by itself an independent risk factor for failure [3, 4, 14, 32]. Also open DAIR without PE exchange is considerably less aggressive in reducing the amount of bacteria in the joint compared to open DAIR with PE exchange. In our cohort, we found no difference in outcome of cases with and without PE exchange in the open DAIR group, which is most likely due to the small case numbers. We didn’t exclude the five cases without PE exchange, which might have caused some heterogeneity in the open DAIR group. Nevertheless, arthroscopic DAIR remained clearly inferior to the open DAIR groups with and without PE exchange. Independent from our data and in accordance with the data presented by Kim et al., we would strongly advocate to exchange the PE liner whenever possible to further reduce the number of bacteria in the joint [17].

Despite several studies on the results of arthroscopic and open DAIR [4, 6, 7, 9, 11, 17, 18, 20, 22, 23, 31–33, 37], there is only the one study of Johns et al. [14] directly comparing outcomes of these two procedures. In line with our study, they presented inferior results with arthroscopic DAIR with a success rate of 16% in arthroscopic vs 45% in open DAIR. In comparison with our data, they presented a lower success rate for the open DAIR procedure (success rate of 45% vs 68%), despite longer intravenously antibiotic therapy (6 weeks vs 2 weeks) as well as longer overall antibiotic treatment duration (4–5 months vs. 3 months) Johns et al. [14] did not provide further information on their antibiotic treatment, thus we can only speculate whether biofilm active antibiotics like Rifampin of Fluoroquinolones have been administered [36]. A close collaboration with ID physicians can reduce the amount, duration and side effects of antibiotic therapy for these complex cases [26]. Our study complements to the study of Johns et al. in terms that their patients follow-up was limited to an average of 2.6 years [14], whereas our average follow-up time after index surgery was 8.4 years. Moreover, we could add some important information on the perioperative management with intensive unit care and surgery timing, which may have influence on the much higher open DAIR success results.

In a multicenter clinical review, Young and colleagues investigated the influence of individual surgeons on surgical outcome in patients with periprosthetic joint infections in TKA over a 15-year period [35]. The results of specialized arthroplasty surgeons were compared to the results of general orthopedic surgeons. Arthroplasty surgeons were showing 2.9 times higher success rates. Even though periprosthetic joint infection is generally an acutely concerning disease, this higher success rate demonstrates that timing and personalizing surgery can be important. In our series only 65% of the open procedures have been performed by senior surgeons, which might in parts explain the low success rate of only 68%. The arthroscopic DAIR procedures were even less likely to be performed by senior physicians (65% vs 31%) which may have led to a bias in disadvantage of the arthroscopic group. During the study period no multidisciplinary team (MDT) dedicated to the treatment of PJI was implemented at our institution. One might speculate if our results would have been better if all procedures would have been performed by senior surgeons or in an MDT setting.

For septic patients, the pathogen reduction is a time-sensitive manner and open DAIR procedure should be carried out as early as possible. In very acute cases, with the threat of septic shock or impending anteperforation, an open procedure should be preferred without delay, even if a new liner is not available. Yet, in stable patients the quality of surgery is more important than speed and thus waiting for a specialized surgical team till the following daytime shift, rather than acute surgery performed by the general orthopedic or trauma surgeon on call, may lead to improved surgical outcomes.

Periprosthetic joint infection is associated with biofilm-generating organisms. *S. aureus*, *S. epidermidis* and *P. aeruginosa* are responsible for approximately 80% [28] of infections, but infections can also be polymicrobial [15, 29]. The acuity and aggressive nature of the infection is influenced by a variety of factors, including the virulence of the microorganism and individual patients characteristics [36]. Kilgus et al. reported that among patients with infected total knee arthroplasty, those with resistant Staphylococci had a higher failure rate compared to those infected with sensitive bacteria (82% versus 11%). However, they did not examine the type of surgical procedure used for treatment of PJI [16]. Nevertheless, microbial definition with optimal personalized antibiotic therapy is a sensitive nonsurgical step in the treatment of PJI and a multimodal approach in collaboration with infectious disease specialists is decisive. Though the differences and application of antibiotics are a substantial part of the treatment, unfortunately the sample size of our cohort is too small for a subgroup analyses or adequate adjustment for the different pathogens with an even broader antibiotic spectrum and resistance patterns. Based on our limited dataset, we cannot draw any significant conclusions. Yet, the antibiotic therapy was in all cases established in close collaboration with an infectious disease specialist and was provided for a period of three months following antibiotic treatment initiation in line with the established PJI protocol.

Our study has several limitations to consider. First, this was a retrospective review of data where decision to perform arthroscopic or open DAIR surgery selection followed no predefined protocol. The selection is therefore highly biased. Yet, our study still shows how a surgeon’s personal preference may be misleading within clinical practice. Second, patients with early postoperative infection may have been more likely to receive an open DAIR procedure due to having a preexisting incision wound. Third, this study involved a relatively small number of patients. Nonetheless, to the best of our knowledge, this is one of the largest cohorts including this specific patient population described in the literature.

## Conclusion

In summary, we recommend open debridement in acute and hematogenous infection, as arthroscopic debridement shows inferior results in PJI recurrence prevention. The extent of debridement with removal of scar tissue and extended synovectomy as well as exchange of PE whenever possible is extremely important. High failure rates are, among others, also owed to the fact that little emphasis lies on patient care performed by the expert surgeon’s sub-specialization and the personalization of treatment. Besides preferring open DAIR, the establishment of a multidisciplinary team, to allow more personalized and specialized treatment, is essential to improve the treatment of this challenging patient population.

## Abbreviations

CRP: C-reactive protein
DAIR: Debridement, antibiotics, and implant retention
EBJIS: European Bone and Joint Infection Society
ID: Infectious disease
OR: Operating room
PJI: Periprosthetic joint infection
PE: Polyethylene
PMN: Polymorphonuclear
WBC: White blood cell count
